# sLe^x^ expression in invasive micropapillary breast carcinoma is associated with poor prognosis and can be combined with MUC1/EMA as a supplementary diagnostic indicator

**DOI:** 10.20892/j.issn.2095-3941.2020.0422

**Published:** 2021-06-15

**Authors:** Yawen Song, Hui Sun, Kailiang Wu, Jianke Lyu, Jingyue Zhang, Feng Gu, Yongjie Ma, Beibei Shen, Chijuan Wang, Xiaojiao Chen, Jing Xu, Weidong Li, Fangfang Liu, Li Fu

**Affiliations:** 1 Department of Breast Cancer Pathology and Research Laboratory, Tianjin Medical University Cancer Institute and Hospital, National Clinical Research Center for Cancer, Key Laboratory of Cancer Prevention and Therapy, Tianjin, Tianjin’s Clinical Research Center for Cancer; Key Laboratory of Breast Cancer Prevention and Therapy, Tianjin Medical University, Ministry of Education; Breast Cancer Innovation Team of the Ministry of Education; State Key Laboratory of Breast Cancer Research, Tianjin 300060, China

**Keywords:** Invasive micropapillary carcinoma, polarity reversal, diagnostic indicator, EMA, sLe^x^

## Abstract

**Objective:**

Mucin 1 (MUC1/EMA) and sialyl Lewis X (sLe^x^) indicate polarity reversal in invasive micropapillary carcinoma (IMPC). The purpose of this study was to evaluate the expression of MUC1/EMA and sLe^x^ and to assess their diagnostic and prognostic value in patients with IMPC.

**Methods:**

The expression of sLe^x^ and MUC1/EMA in 100 patients with IMPC and a control group of 89 patients with invasive ductal carcinoma not otherwise specified (IDC-NOS) were analyzed with IHC. Fresh tumor tissues were collected from patients with IMPC or IDC-NOS for primary culture and immunofluorescence analysis.

**Results:**

The rate of nodal metastasis was higher in patients with IMPC than those with IDC-NOS, and IMPC cells tended to express more sLe^x^ and MUC1/EMA in the cytomembranes (the stroma-facing surfaces of the micropapillary clusters) than IDC-NOS cells. In IMPC, high cytomembrane expression of sLe^x^, but not MUC1/EMA, indicated poor prognosis. In addition, among the 100 patients with IMPC, 10 patients had sLe^x^+/EMA– expression patterns, and 8 patients had sLe^x^–/EMA+ expression patterns. The primary IMPC cells were suspended, non-adherent tumor cell clusters, whereas the primary IDC cells were adherent tumor cells. Immunofluorescence analysis showed that MUC1/EMA and sLe^x^ were co-expressed on the cytomembranes in IMPC cell clusters and in the cytoplasm in IDC-NOS cells.

**Conclusions:**

sLe^x^ can be used as a prognostic indicator and can be combined with MUC1/EMA as a complementary diagnostic indicator to avoid missed IMPC diagnosis.

## Introduction

Invasive micropapillary carcinoma (IMPC) is a type of breast cancer accounting for approximately 7% of all breast cancer cases^1^. IMPC can be characterized by the morphotype of mulberry-like cell clusters, which lack central vascular bundles and are surrounded by a clear interstitial space^2^. IMPC can be recognized by the typical “inside-out” growth pattern, which indicates the “polarity reversal” of IMPC cells^3^. Cancer cells with polarity reversal show high metastatic potential and can be recognized by the presence of an inside-out expression pattern through MUC1/EMA or sLe^x^ staining^4–7^.

MUC1, also known as EMA^8,9^, is a high molecular weight (> 400,000) type I transmembrane glycoprotein mainly distributed in glandular epithelial cells. MUC1/EMA with high glycosylation (glycosylation > 50%) is composed of core peptides and sugar chains, most of which are attached through O-linked glycosylation. The core peptides of MUC1/EMA contain intracellular, transmembrane, and extracellular regions.

The carbohydrate ligand sialyl Lewis X (sLe^x^) is an adhesion molecule expressed on the surfaces of human leukocytes and various cancer cells. It is the most important ligand for selectin, particularly E-selectin, which is expressed on the surfaces of endothelial cells^10^. sLe^x^ and MUC1/EMA are associated with the reversal of cell polarity, which enhances the metastatic potential of breast cancer, particularly lymph node metastasis of IMPC^7,11^.

Although few studies have proposed a relationship between sLe^x^ and breast cancer prognosis^12–14^, no detailed analysis has been performed on the expression of sLe^x^ in the invasive micropapillary structure and its prognostic value for IMPC. Several studies have shown that MUC1/EMA can be a carrier of sLe^x^, and sLe^x^ in turn is an epitope of MUC1/EMA^15,16^. Therefore, this study evaluated the distribution of sLe^x^ and MUC1/EMA expression in IMPC and analyzed their prognostic value.

## Materials and methods

### Case selection

One hundred cases of breast IMPC diagnosed in the Department of Breast Pathology of Tianjin Medical University Cancer Institute and Hospital between January 2007 and December 2008 were selected, and contiguous slices were made. Eighty-nine cases of breast IDC-NOS diagnosed in the same period were randomly selected as a control group. Of the 100 cases of breast IMPC, 94 cases were of mixed type, and 6 cases were of pure type. Mixed type IMPC comprises both IMPC and IDC-NOS components. The median age of patients in the IMPC group at diagnosis was 52 years (range 28–89), and the median age of patients in the IDC-NOS group was 50 years (range 28–80). The follow-up time for the 2 groups was 1–100 months (median follow-up time of 63 months). A total of 3 IMPC and 3 IDC-NOS fresh tumor tissues were collected for primary culture. The study was approved by the Ethics Committee of Tianjin Medical University Cancer Institute and Hospital, and informed consent was obtained from all patients.

### Immunohistochemistry

Four-micrometer serial whole-tissue sections were cut from the archived formalin-fixed, paraffin-embedded tissue blocks, dewaxed, and subsequently rehydrated with xylene and graded alcohol washes. Antigen retrieval (sLe^x^) was performed in a pressure cooker in citrate buffer (pH 6.0) for 2 min 30 s, and EMA was performed in EDTA (pH 9.5). The sections were treated with 3% hydrogen peroxide for 10 min to block endogenous peroxidase activity and then incubated with normal goat serum for 10 min to eliminate nonspecific background staining. Thereafter, primary antibodies to sLe^x^ (BD Biosciences, #551344, monoclonal, 1:150 dilution, CA, USA) or EMA (ZSGB-bio, #ZM-0095, monoclonal, Beijing, China) were incubated with the samples at 4 °C overnight. Antigen was sequentially detected with secondary biotin-labeled antibody and peroxidase-conjugated streptavidin. The chromogen was 3,3-diaminobenzidine. The sections were counterstained with hematoxylin.

### Immunohistochemistry scoring

**Figure 1:**
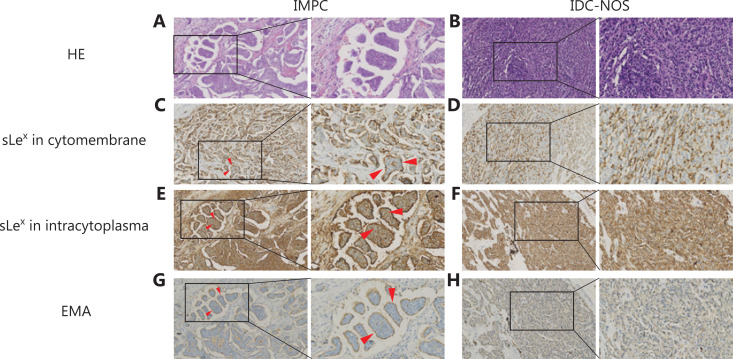
Immunohistochemical expression of sLe^x^ and MUC1/EMA in breast IMPC and IDC-NOS. (A) IMPC consists of tumor cell clusters of epithelial cells surrounded by gaps [hematoxylin and eosin (HE) staining]. (B) Representative microphotographs of IDC-NOS (HE). Immunohistochemistry confirming the expression of sLe^x^ on the cytomembranes in IMPC cell clusters (C) and IDC-NOS cells (D). The expression of sLe^x^ within the cytoplasm in IMPC cell clusters (E) and IDC-NOS cells (F). EMA immunostaining showing the typical polarity reversal growth pattern of IMPC (G) and the normal pattern in IDC-NOS tumor cells (H) (200×, 400×, indicated by the red arrow).

MUC1/EMA and sLe^x^ expression was localized to the cytomembrane (the stroma-facing surfaces of the cell clusters) or within the cytoplasm (**Figure 1**). Only IMPC component staining in mixed type IMPC was recorded. For the IDC-NOS group, we also recorded the staining of the cytomembrane or cytoplasm.

SLe^x^ immunostaining was assessed through light microscopy by 2 independent experienced pathologists (Li Fu and Fangfang Liu). Both pathologists reevaluated the staining and reached a conclusion by consensus. The antibody staining patterns were scored and calculated as the average of I × P, where I is the intensity of staining (0, 1, 2, or 3), and P is the percentage of positive tumor cells (0%–100%). The intensity of staining was scored as no staining (0), low intensity (1), moderate intensity (2), or high intensity (3). The sLe^x^ expression was categorized as negative when the score was below 2.5 or as high when the score was above 60^17^.

MUC1/EMA immunoreactivity was evaluated in a semiquantitative manner^18^. Staining intensity was classified as negative (0), low (1), moderate (2), or high (3). The percentage of positive cells was graded as follows: 0: no positive cells; 1: positive staining in less than 5% of cells; 2: positive staining in 5%–30% of cells; 3: positive staining in 30%–60% of cells; and 4: positive staining in more than 60% of cells. A semiquantitative scoring system was applied by multiplying the intensity and percentage of MUC1/EMA-positive cells. Total scores were grouped as follows: 0 (negative reaction); 1 to 6 (weak reaction); and 7 to 12 (strong reaction). A score of 0 to 6 was regarded as low expression, and a score greater than 6 was regarded as high expression.

### Primary tumor cell culture

IMPC and IDC-NOS tissue samples were collected for primary cell culture. The samples were washed twice in normal saline and cut into small pieces (< 1 mm). Then 1.5 mL cell dispersing enzyme EZ solution was added, and samples were digested at 37 °C for 2 h on an oscillator. After the digestion was completed (i.e., many cells were observed to be separated from the tissue under a microscope), the cells were filtered with a 308 µM nylon net. The cell precipitates were re-suspended in 3 mL modified medium, then transferred to a 6 cm dish. The primary IMPC and IDC-NOS tumor cells were cultured in DMEM/F12 medium, which was supplemented with 5% horse serum, 10 µg/mL insulin, 20 ng/mL Maxime EGF, 0.5 µg/mL hydrocortisone, 100 ng/mL cholera toxin, 10 µM RhoA kinase inhibitor (Y - 27632), 4 mM L-glutamine, 1 mM pyruvate, 0.05% bovine pituitary extract, and 1% penicillin-streptomycin. Then the cells were cultured in an incubator at 37 °C, with 5% CO_2_.

### 3D culture and immunofluorescence staining of primary tumor cells

The primary IMPC and IDC-NOS cell suspensions were mixed with collagen A, B, and C (Nitta Gelatin, Osaka, Japan) at 8:1:1 on ice, and then 30 µL drops were deposited on a glass cover slide coated with fibronectin, which was placed on a 6-well plate (Corning, NY, USA). After culture in a 37 °C, 5% CO_2_ incubator for 30 min, the collagen droplets were solidified, and 2 mL medium was added for long-term culture. The IMPC and IDC-NOS cells were then fixed in collagen droplets with 4% paraformaldehyde and permeabilized with 0.2% Triton X-100. Antibodies to sLe^x^ (BD Biosciences, #551344, monoclonal, 1:150 dilution, CA, USA) or EMA (ZSGB-bio, #ZM-0095, monoclonal, Beijing, China) were then incubated with the samples at 4 °C overnight. Secondary antibody was incubated at room temperature for 1 h. DAPI (Solarbio, Beijing, China) was used to stain the nuclei, and images were obtained through confocal microscopy.

### Statistical analysis

Statistical analyses were performed in SPSS Statistics 23.0 (IBM Corporation, Chicago, IL, USA). A Spearman rank correlation test was performed to assess the relationships among MUC1/EMA and sLe^x^ expression and clinicopathological characteristics. The Kaplan-Meier method was used to construct survival curves (disease-free survival, DFS; overall survival, OS), which were compared with the log-rank test. The Cox proportional hazard model was used for univariate and multivariate analyses. A *P*-value < 0.05 was considered statistically significant.

## Results

### Clinicopathological characteristics

Compared with patients with IDC-NOS, patients with IMPC had a significantly higher frequency of lymph node metastasis (77.0% *vs.* 51.7%; *P* < 0.001) and lymphovascular invasion (LVI, 28.0% *vs.* 3.4%; *P* < 0.001), and a higher risk of local recurrence and metastasis (38.0% *vs.* 16.3%; *P* < 0.001). There was no significant difference in patient age, tumor size, distribution of histological grades, ER status, PR status, or HER-2 status between the IMPC and IDC-NOS groups (*P* > 0.05) (**Table 1**).

**Table 1:** Clinicopathological characteristics of patients with IMPC and IDC-NOS

Characteristics	IMPC *n* (%)	IDC-NOS *n* (%)	*P*
Age			0.111
≤ 52	39 (39.0)	45 (50.6)	
> 52	61 (61.0)	44 (49.4)	
Tumor size			0.182
T1–T2	86 (86.0)	82 (92.1)	
T3–T4	14 (14.0)	7 (7.9)	
Histological grade			0.897
I	13 (13.0)	6 (6.7)	
II	64 (64.0)	69 (77.5)	
III	23 (23.0)	14 (15.7)	
ER			0.418
Negative	38 (38.0)	39 (43.8)	
Positive	62 (62.0)	50 (56.2)	
PR			0.607
Negative	39 (39.0)	38 (42.7)	
Positive	61 (61.0)	51 (57.3)	
*HER2			0.414
Negative	66 (66.0)	*53 (60.2)	
Positive	34 (34.0)	*35 (39.8)	
Lymphovascular invasion			< 0.001*
Negative	72 (72.0)	86 (96.6)	
Positive	28 (28.0)	3 (3.4)	
pTNM			< 0.001*
I	4 (4.0)	14 (15.7)	
II	57 (57.0)	61 (68.6)	
III	39 (39.0)	14 (15.7)	
Lymph node metastasis			< 0.001*
Negative	23 (23.0)	43 (48.3)	
Positive	77 (77.0)	46 (51.7)	
sLe^x^ in cytomembrane			< 0.001*
Low	60 (60.0)	82 (92.1)	
High	40 (40.0)	7 (7.9)	
sLe^x^ in cytoplasm			< 0.001*
Low	67 (67.0)	83 (93.3)	
High	33 (33.0)	6 (6.7)	
EMA in cytomembrane			< 0.001*
Low	62 (62.0)	88 (98.8)	
High	38 (38.0)	1 (1.1)	
EMA in cytoplasm			< 0.001*
Low	87 (87.0)	89 (100.0)	
High	13 (13.0)	0 (0)	
Recurrence or metastasis			0.002*
No	62 (62.0)	76 (85.4)	
Yes	38 (38.0)	13 (14.6)	

IMPC, invasive micropapillary carcinoma; IDC-NOS, invasive ductal carcinoma, non-special type; sLe^x^, sialylated Lewis glycoprotein X; EMA, epithelial membrane antigen. *P* < 0.05, calculated with chi-square test. *There is a IDC-NOS case lacking information about HER2.

### IHC expression of sLe^x^ and MUC1/EMA in IMPC and IDC

The immunohistochemical results showed that MUC1/EMA and sLe^x^ were mainly expressed on the cytomembrane in IMPC (**Figure 1G and 1C**). The expression of MUC1/EMA and sLe^x^ in IDC-NOS was mainly within the cytoplasm (**Figure 1H and 1F**). Both cytomembrane and intracytoplasmic expression of sLe^x^ or MUC1/EMA was much more frequently detected in IMPC sections than in IDC-NOS sections (*P* < 0.001, *P* < 0.001 for cytomembrane expression and *P* < 0.001, *P* < 0.001 for intracytoplasmic expression) (**Table 3**). However, some IMPC cases showed either negative MUC1/EMA or sLe^x^ expression on the cytomembrane, including 10 cases displaying sLe^x^+/EMA– expression and 8 cases displaying sLe^x^–/EMA+ expression (**Table 2**). The high expression of sLe^x^ within the cytoplasm in patients with IMPC was associated with high histological grade and ER expression (*P* < 0.001, *P* = 0.041, *P* = 0.048), and patients with IMPC with high expression of sLe^x^, either on the cytomembrane or in the cytoplasm, appeared to have a higher frequency of tumor recurrence and metastasis (*P* < 0.001), whereas patients with high expression of MUC1/EMA did not. In addition, patients with IMPC with high expression of MUC1/EMA on the cytomembrane were mostly older than 52 years and had pTNM stage I or II disease (*P* = 0.004; *P* = 0.029). Patients with HER2 positive expression tended to express more MUC1/EMA within the cytoplasm in IMPC tumor cells than those with negative HER2 expression (*P* = 0.025) (**Table 3**).

**Table 2:** Crosstabulation of EMA and sLe^x^ expression in IMPC and their correlation

Characteristics	EMA in cytomembrane			EMA in cytoplasm		
	Negative *n* (%)	Positive *n* (%)	*P* value	Negative *n* (%)	Positive *n* (%)	*P* value
sLe^x^ in cytomembrane			0.328			
Negative	0 (0.0)	8 (8.9)				
Positive	10 (100.0)	82 (91.1)				
sLe^x^ in cytoplasm						0.288
Negative				6 (17.6)	18 (27.3)	
Positive				28 (82.4)	48 (72.7)	

IMPC, invasive micropapillary carcinoma; IDC-NOS, invasive ductal carcinoma, non-special type; sLe^x^, sialylated Lewis glycoprotein X; EMA, epithelial membrane antigen. *P* < 0.05, calculated with chi-square test.

**Table 3:** EMA and sLe^x^ expression in IMPC and their correlation with clinicopathologic parameters

Characteristics	sLe^x^ in cytomembrane			sLe^x^ in cytoplasm			EMA in cytomembrane			EMA in cytoplasm		
	Low (%)	High (%)	*P*	Low (%)	High (%)	*P*	Low (%)	High (%)	*P*	Low (%)	High (%)	*P*
Age			0.318			0.073			0.004*			0.209
≤ 52	21 (35.0)	18 (45.0)		22 (32.8)	17 (51.5)		31 (50.0)	8 (21.1)		36 (41.4)	3 (23.1)
> 52	39 (65.0)	22 (55.0)		45 (67.2)	16 (48.5)		31 (50.0)	30 (78.9)		51 (58.6)	10 (76.9)
Tumor size			0.815			0.147			0.436			0.878
T1–T2	52 (86.7)	34 (85.0)		60 (89.6)	26 (78.8)		52 (83.9)	34 (89.5)		75 (86.2)	11 (84.6)
T3–T4	8 (13.3)	6 (15.0)		7 (10.4)	7 (21.2)		10 (16.1)	4 (10.5)		12 (13.8)	2 (15.4)
Histological grade			0.170			0.041*			0.782			0.177
I	11 (18.3)	2 (5.0)		12 (17.9.)	1 (3.0)		7 (11.3)	6 (15.8)		13 (15.0)	0 (0.0)
II	36 (60.0)	28 (70.0)		42 (62.7)	22 (66.7)		41 (66.1)	23 (60.5)		55 (63.2)	9 (69.2)
III	13 (21.7)	10 (25.0)		13 (19.4)	10 (30.3)		14 (22.6)	9 (23.7)		19 (21.8)	4 (30.8)
ER			0.181			0.048*			0.146			0.567
Negative	26 (43.3)	12 (30.0)		30 (44.8)	8 (24. 2)		27 (43.5)	11 (28.9)		34 (39.1)	4 (30. 8)
Positive	34 (56.7)	28 (70.0)		37 (55.2)	25 (75.8)		35 (56.5)	27 (71.1)		53 (60.9)	9 (69.2)
PR			0.134			0.213			0.620			0.966
Negative	27 (45.0)	12 (30.0)		29 (43.3)	10 (30.3)		23 (37.1)	16 (42.1)		34 (39.1)	5 (38.5)
Positive	33 (55.0)	28 (70.0)		38 (56.7)	23 (69.7)		39 (62.9)	22 (57.9)		53 (60.9)	8 (61.5)
HER2			0.864			0.091			0.368			0.025*
Negative	40 (66.7)	26 (65.0)		48 (71.6)	18 (54.5)		43 (69.4)	23 (60.5)		61 (70.1)	5 (38.5)
Positive	20 (33.3)	14 (35.0)		19 (28.4)	15 (45.5)		19 (30.6)	15 (39.5)		26 (29.9)	8 (61.5)
Lymph node metastasis			0.997			0.134			0.680			0.637
N0	23 (39.0)	14 (35.0)		26 (39.4)	11 (33.3)		23 (37.7)	14 (36.8)		32 (37.2)	5 (38.5)
N1	17 (28.8)	17 (42.5)		24 (36.4)	10 (30.3)		22 (36.1)	12 (31.6)		30 (34.9)	4 (30.8)
N2	12 (20.3)	2 (5.0)		10 (15.2)	4 (12.1)		8 (13.1)	6 (15.8)		10 (11.6)	4 (30.8)
N3	7 (11.9)	7 (17.5)		6 (9.0)	8 (24.3)		8 (13.1)	6 (15.8)		14 (16.3)	0 (0.0)
Lymphovascular invasion			0.148			0.910			0.869			0.370
Negative	40 (66.7)	32 (80.0)		48 (71.6)	24 (72.7)		45 (72.6)	27 (71.1)		64 (73.6)	8 (61.5)
Positive	20 (33.3)	8 (20.0)		19 (28.4)	9 (27.3)		17 (27.4)	11 (28.9)		23 (26.4)	5 (38.5)
pTNM stage			0.434			0.328			0.029*			0.794
I	4 (6.9)	0 (0.0)		4 (6.2)	0 (0.0)		1 (1.6)	3 (8.1)		4 (4.7)	0 (0.0)
II	32 (55.2)	24 (60.0)		37 (56.9)	19 (57.6)		32 (52.5)	24 (64.9)		48 (56.5)	8 (61.5)
III	22 (37.9)	16 (40.0)		24 (36.9)	14 (42.4)		28 (45.9)	10 (27.0)		33 (38.8)	5 (38.5)
Recurrence or metastasis			< 0.001*			< 0.001*			0.563			0.720
No	43 (79.6)	14 (36.8)		47 (78.3)	10 (31.2)		34 (59.6)	23 (65.7)		49 (61.2)	8 (66.7)
Yes	11 (20.4)	24 (63.2)		13 (21.7)	22 (68.8)		23 (40.4)	12 (34.3)		31 (38.8)	4 (33.3)

*P < 0.05 was considered statistically significant. ER, estrogen receptor; PR, progesterone receptor; HER-2, human epidermal factor receptor 2; IMPC, invasive micropapillary carcinoma; IDC-NOS, invasive ductal carcinoma, non-special type; sLe^x^, sialylated Lewis glycoprotein X; EMA, epithelial membrane antigen. *P* < 0.05, calculated with chi-square test.

### Expression of sLe^x^ and MUC1/EMA in IMPC and IDC primary cells

**Figure 2:**
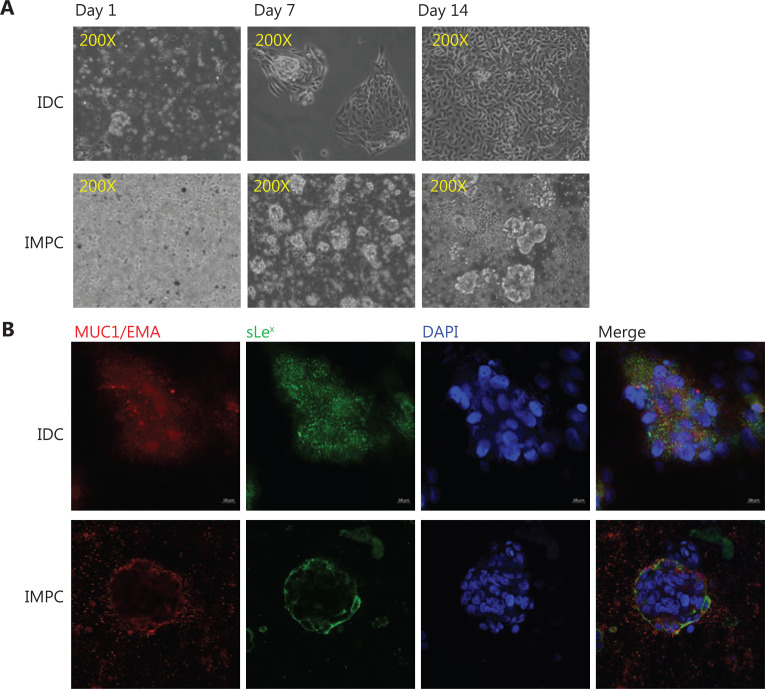
Immunofluorescence expression of sLe^x^ and MUC1/EMA in breast IMPC and IDC-NOS. (A) Cell morphology of primary cultured IMPC and IDC-NOS cells. Primary IMPC cells are suspended, non-adherent tumor cell clusters; primary IDC cells are adherent tumor cells (200×). (B) Immunofluorescence confirming the expression of MUC1/EMA and sLe^x^ on the cytomembranes in IMPC cell clusters and IDC-NOS cells (630×).

The primary IMPC cells were suspended, non-adherent tumor cell clusters, whereas the primary IDC cells were adherent tumor cells (**Figure 2A**). Immunofluorescence showed that MUC1/EMA and sLe^x^ were mainly expressed on the cytomembranes in IMPC cell clusters (**Figure 2B**), whereas the expression of MUC1/EMA and sLe^x^ in IDC-NOS cells was mainly within the cytoplasm (**Figure 2B**). Moreover, immunofluorescence indicated that MUC1/EMA and sLe^x^ were co-expressed on the cytomembranes in IMPC cell clusters (**Figure 2B**), in agreement with the results of IHC staining of MUC1/EMA and sLe^x^.

### Prognostic value of sLe^x^ and MUC1/EMA expression in IMPC

**Figure 3:**
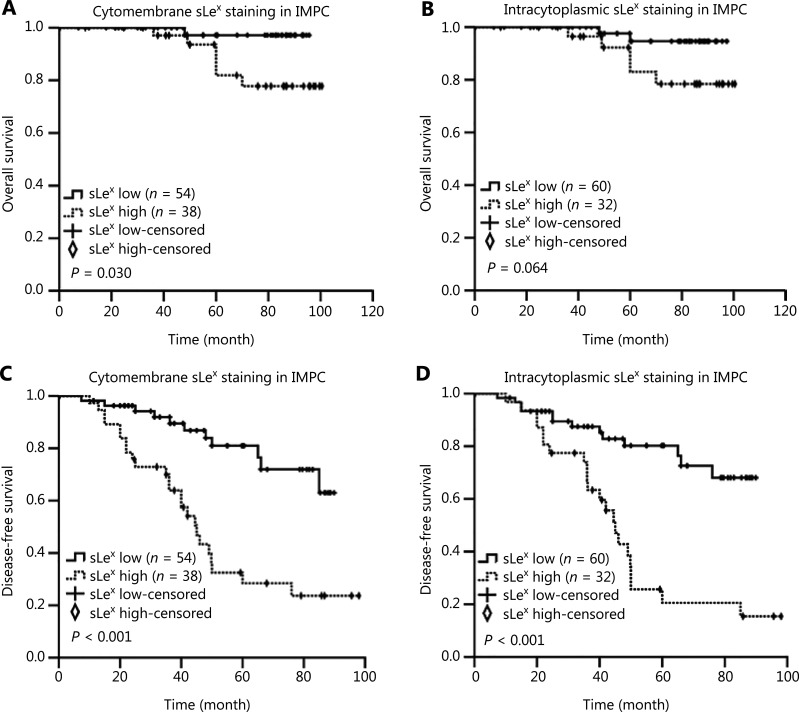
Kaplan-Meier curves showing the prognostic value of sLe^x^ expression in patients with IMPC. Kaplan-Meier curve for (A) OS and (C) DFS, based on the cytomembrane expression of sLe^x^. (B) OS and (D) DFS, based on the intracytoplasmic expression of sLe^x^. *P*-values were calculated with the log-rank test. Eight patients were lost to follow-up.

**Figure 4:**
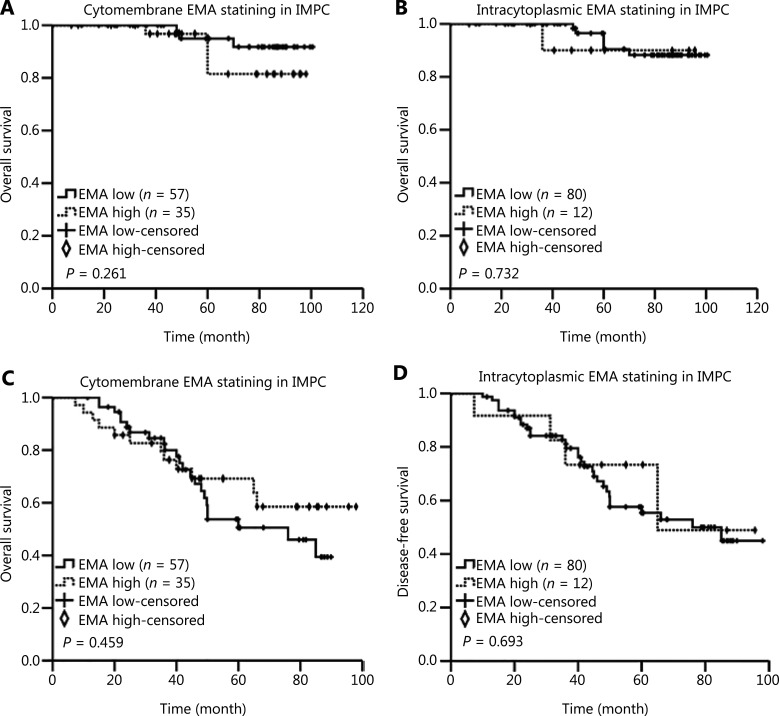
Kaplan-Meier curves showing the prognostic value of EMA expression in patients with IMPC. Kaplan-Meier curve for (A) OS and (C) DFS, based on the cytomembrane expression of EMA. (B) OS and (D) DFS, based on the intracytoplasmic expression of EMA. *P*-values were calculated with the log-rank test. Eight patients were lost to follow-up.

Kaplan-Meier survival curves revealed that the high expression of sLe^x^ on the cytomembranes in the IMPC tumor cell masses was associated with poor prognosis in terms of OS and DFS in patients with IMPC (*P* = 0.030 and *P* < 0.001, **Figure 3A and 3C**). High sLe^x^ expression within the cytoplasm in IMPC was indicative of relatively short DFS in patients with IMPC (*P* < 0.001, **Figure 3D**). Moreover, high expression of MUC1/EMA in all parts of the cell in the IMPC group had no significant association with OS and DFS (**Figure 4A–4D**). Univariate Cox proportional hazard model analysis confirmed that histological grade, lymph node metastasis, and high expression of sLe^x^ on the cytomembrane, but not in the cytoplasm, were prognostic factors for DFS in patients with IMPC (**Table 4**). Multivariate Cox regression analysis showed that lymph node metastasis and high expression of sLe^x^ on the cytomembrane were independent predictors of poor DFS in patients with IMPC (*P* = 0.014, HR = 1.486, 95%CI = 1.084–2.037; *P* = 0.025, HR = 3.099, 95%CI = 1.150–8.352) (**Table 5**).

**Table 4:** Univariate analysis of patients with breast IMPC

Factors	DFS			OS		
	HR	95%CI	*P*	HR	95%CI	*P*
Age (≤ 52 *vs.* > 52)	0.717	0.361–1.422	0.341	0.708	0.158–3.166	0.652
Tumor size					
(T1–T2 *vs.* T3–T4)	1.557	0.705–3.436	0.273	0.770	0.093–6.400	0.809
Histological grade					
(I *vs.* II *vs.* III)	1.947	1.086–3.489	0.025*	0.376	0.107–1.318	0.126
ER (negative *vs.* positive)	1.567	0.752–3.264	0.231	3.489	0.420–29.010	0.248
PR (negative *vs.* positive)	0.859	0.439–1.681	0.657	0.759	0.170–3.392	0.718
HER2 (negative *vs.* positive)	1.100	0.562–2.152	0.781	0.642	0.124–3.314	0.597
sLe^x^ in cytomembrane	4.137	2.019–8.478	< 0.001*	7.247	0.872–60.200	0.067
sLe^x^ in cytoplasm	4.207	2.101–8.422	< 0.001*	4.114	0.798–21.211	0.091
EMA in cytomembrane	0.769	0.382–1.548	0.462	2.278	0.512–10.252	0.278
EMA in cytoplasm	0.812	0.286–2.302	0.695	1.442	0.173–12.011	0.735
Chemotherapy	0.993	0.300–3.286	0.991	0.445	0.052–3.813	0.460
Radiotherapy	2.016	0.987–4.117	0.054	4.277	0.783–23.376	0.094
Endocrine therapy	0.658	0.295–1.466	0.306	0.836	0.153–4.579	0.837
Lymph nodes					
(N0 *vs.* N1 *vs.* N2 *vs.* N3)	1.480	1.106–1.981	0.008*	1.756	0.917–3.362	0.090

*P < 0.05 was considered statistically significant. DFS, disease-free survival; OS, overall survival; ER, estrogen receptor; PR, progesterone receptor; HER-2, human epidermal factor receptor 2; IMPC, invasive micropapillary carcinoma; IDC-NOS, invasive ductal carcinoma, non-special type; sLe^x^, sialylated Lewis glycoprotein X; EMA, epithelial membrane antigen. *P* < 0.05, calculated with the log-rank test.

**Table 5:** Multivariate analysis of patients with breast IMPC

Factors	DFS			OS		
	HR	95%CI	*P*	HR	95%CI	*P*
Age (≤ 52 *vs.* > 52)	0.762	0.370–1.569	0.461	0.798	0.163–3.910	0.781
Tumor size					
(T1–T2 *vs.* T3–T4)	1.007	0.423–2.400	0.987	0.977	0.103–9.321	0.984
Histological grade					
(I *vs.* II *vs.* III)	1.492	0.790–2.821	0.218	0.305	0.071–1.318	0.112
sLe^x^ in cytomembrane	3.099	1.150–8.352	0.025*	5.740	0.467–70.562	0.172
sLe^x^ in cytoplasm	1.526	0.569–4.092	0.401	1.324	0.138–12.657	0.808
Lymph nodes					
(N0 *vs.* N1 *vs.* N2 *vs.* N3)	1.486	1.084–2.037	0.014*	1.470	0.718–3.011	0.292

*P < 0.05 was considered statistically significant. DFS, disease-free survival; OS, overall survival; IMPC, invasive micropapillary carcinoma; IDC-NOS, invasive ductal carcinoma, non-special type; sLe^x^, sialylated Lewis glycoprotein X; EMA, epithelial membrane antigen. *P* < 0.05, calculated with the log-rank test.

## Discussion

IMPC was listed in the 2003 WHO Histological Classification of Breast Tumors as a subtype of invasive carcinoma^19^. This tumor can be identified by the characteristic feature of an “inside-out” growth pattern, suggesting a reversal in cell polarity. IMPC cancer cells with reversed polarity have more aggressive biological behaviors, particularly a striking propensity for lymphatic invasion and nodal metastasis, regardless of the proportion of their IMPC component^20–25^. However, the underlying mechanism remains unclear. The metastasis of breast cancers is highly dependent on the interaction of adhesion molecules in cell-cell and/or cell-matrix contexts, and alterations in glycosylation patterns on the cancer cell surface can drive cancer metastasis though ligand-receptor-mediated interactions^26^.

Acs^3^ has proposed that the expression of MUC1/EMA on the periphery of IMPC tumor cell clusters may contribute to tumor progression and lymphatic metastasis. However, our findings suggest that the high expression of MUC1/EMA on the cell membrane and within the cytoplasm is not associated with prognosis in patients with IMPC or IDC-NOS. This finding may be because MUC1 is mainly involved in the transcriptional activation of several oncogenes, despite MUC1/EMA’s high molecular weight and multiple functions driving breast cancer progression^27–29^. It cannot be used as a dedicated ligand by vascular and lymphatic endothelial cells to promote the metastasis of cancer cells.

sLe^x^, also known as CD15s, is a sialylated tetrasaccharide structure displayed at the cell surface, on both glycoproteins and glycolipids. sLe^x^ is present on the surfaces of leukocytes, and it plays an important role in cell-cell interaction. sLe^x^ has been proposed to participate in regulating the adhesion between cancer cells and endothelial cells. As the most important glycan ligand for E-selectin (which is expressed on the surfaces of endothelial cells)^10,30–32^, sLe^x^ is present on the surfaces of many types of cancer cells, including breast IMPC cells^7,12–16^, and it plays an important role in the extravasation of cancer cells from the blood or lymph vessels, thereby promoting the migration of cancer cells to distant organs. Miyara has demonstrated that CD15s is highly specific for activated, terminally differentiated, and most suppressive FOXP3^high^ effector Treg cells, thus suggesting that cancer cells can mimic the behavior of immune cells to escape immunological surveillance and establish new metastatic foci^33^. Therefore, overexpression of sLe^x^ has frequently been reported to correspond with poorer outcomes and malignancy recurrence^10,11,34^. In addition, glycosyltransferases, which produce sLe^x^ exclusively by fucosylation of sialylated LacNAc in humans, have been reported to increase EMT and migration ability in breast and hepatic cancers^35–37^.

In this study, we indeed found that both cytomembrane and intracytoplasmic expression of sLe^x^ were much more frequently detected in IMPC than in IDC-NOS, and the high expression of sLe^x^ on the cell membrane and within the cytoplasm was identified as a factor indicating poor prognosis and DFS, but not OS, in patients with IMPC; however, patients with IMPC with high expression of sLe^x^ showed a relatively shorter OS time. We attributed the lack of significance in our results to the small sample size studied. Moreover, univariate and multivariate Cox regression analyses showed that high expression of sLe^x^ on the cytomembrane was an independent predictor of poor DFS in patients with IMPC, but the expression of sLe^x^ in patients with IDC-NOS was not associated with DFS or OS; this finding may be explained by the observation that cells overexpressing sLe^x^ in IDC-NOS did not exhibit polarity reversal.

MUC1/EMA has long been used as an auxiliary marker for the clinical diagnosis of IMPC^38–41^. We found that some IMPC cases showed negative MUC1/EMA or sLe^x^ expression on the cytomembrane, including 10 cases with sLe^x^+/EMA– expression and 8 cases with sLe^x^–/EMA+ expression, thus indicating that a combined pathological diagnosis of IMPC with MUC1/EMA and sLe^x^ assessment is needed to avoid missed diagnosis. In contrast to sLe^x^, the expression of MUC1/EMA on the cytomembrane or in the cytoplasm, was not associated with patient prognosis in IMPC. We speculate that MUC1/EMA may not be the only glycoprotein on the cytomembranes in IMPC clusters that can be modified by sLe^x^. MUC1/EMA is the major carrier of sLe^x^ in serous borderline ovarian cancer, adenocarcinoma, and micropapillary bladder urothelial carcinoma, whereas sLe^x^ is an epitope of MUC1/EMA^15,16^. Our IHC results suggest that the role of sLe^x^ in promoting IMPC cluster transfer to distant metastatic sites is not completely dependent on MUC1/EMA—an aspect that might mainly be due to the interaction of adhesion molecules on endothelial cells. sLe^x^, which is overexpressed on the cytomembranes in IMPC cell clusters, interacts with E-selectin (which is highly expressed on the surfaces of lymphatic endothelial cells); this mechanism may be partially responsible for the lymphatic invasion and lymph node metastasis of IMPC cell clusters. In addition, the sugar chain of sLe^x^ contains large amounts of sialic acid, which makes cell surfaces negatively charged. The fucose portion of sLe^x^ specifically binds the C-type lectin-like region of E-selectin, both of which are negatively charged and form strong ionic bonds mediated by calcium ions, thus helping cancer cells pass through the basement membranes of vascular or lymphatic endothelial cells, and promoting tumor cell invasion and metastasis^42–45^. However, in the immunological microenvironment of IMPC, sLe^x^ may evade the cytotoxicity of immune cells mainly through its interaction with NK cells. The sialic acid component of sLe^x^ can directly mask tumor antigens, thus decreasing the sensitivity of tumors to NK cells^46–48^. These reasons may explain why sLe^x^ promotes tumor cell metastasis.

Our results showed that the overexpression of sLe^x^ on the cytomembranes in cell clusters was an independent negative prognostic factor in patients with IMPC but not those with IDC-NOS. This result differed from Sozzani’s findings that sLe^x^ expression is not a prognostic factor in patients with breast cancer^49^. This may be the reason that sLe^x^ is closely associated with the polarity reversal pattern in patients with IMPC, but Sozzani found that sLe^x^ expression was not a prognostic factor in patients with breast cancer^49^, we thought the explanation was that IMPC (a special type of breast cancer) had a special structure (polarity reversal pattern) which IDC-NOS (ordinary breast cancer) had not, and sLe^x^ was closely associated with the polarity reversal pattern in patients with IMPC.

In contrast to the use of MUC1/EMA for tissue diagnosis, the use of sLe^x^, which has a lower molecular weight and is of prognostic value, can enable more convenient serum-based diagnosis and may serve as an index for IMPC glycopeptide monitoring, thus potentially improving the sensitivity and specificity of diagnostic targets and providing strong support for glycopeptide detection. In addition, the metastasis of IMPC clusters may be prevented by blocking the interaction between E-selectin and sLe^x^ or by decreasing the expression of E-selectin, thus providing a potential target for targeted IMPC therapy. Therefore, sLe^x^ may be an important supplementary diagnostic marker of IMPC, and the overexpression of sLe^x^ on the cytomembranes in cell clusters may serve as an independent factor indicating poor prognosis in patients with IMPC. Therapies targeting sLe^x^ or the interaction of sLe^x^ and E-selectin may provide benefits for patients with IMPC in the future.

Because we were unable to find a suitable cell model to represent IMPC cells to perform a series of experiments, we attempted to establish a research model of IMPC through primary culture. Unfortunately, the primary tumor cells, particularly IMPC cell clusters, were difficult to culture, because the primary tumor cell clusters scarcely proliferated. Constructing a suitable cell model to study the mechanisms of IMPC clusters will be the primary task in our future research.
